# Umbilical Cord Blood and UC-MSCs Combined with Low-Dose Immunosuppressant in the Treatment of Elderly Patients with Pure Red Cell Aplastic: A Case Series

**DOI:** 10.2174/011574888X290378240424075002

**Published:** 2024-05-02

**Authors:** Wei-Wei Zhu, Sujing Zhuang, Zhe Yu, Xin Li, Tian-Jie Han, Yue Ma, Li-Jun Li, Zhi-Rui Zhao

**Affiliations:** 1 Department of Hematology, Shandong Provincial Third Hospital, Jinan, 250031, China;; 2 Shandong Qilu Stem Cell Engineering Co.LTD, Jinan, 250102, China

**Keywords:** Pure red cell aplasia, cord blood, mesenchymal stem cells, cyclosporine A, immunotherapy, elderly patients

## Abstract

**Introduction:**

At present, cyclosporine (CsA) is the first-line treatment for Pure Red Cell Aplasia (PRCA), but CsA administration can be associated with a number of side effects due to its high toxicity. Therefore, it is urgent to explore a safe and effective treatment for elderly patients who cannot be treated with conventional doses of CsA, especially those with multiple complications. Allogeneic Stem Cell Transplantation (ASCT) for PRCA is a promising treatment, but reports of using umbilical cord blood (UCB) are very rare.

**Case Presentation:**

In this report, UCB and umbilical cord mesenchymal stem cells (UC-MSCs) combined with low-dose CsA (1-3mg/kg/d) were used to treat 3 elderly patients who were diagnosed with PRCA combined with multiple complications in heart, lung, and renal. The treatments were successful without complications, and 12 months after stem cell infusion, the blood tests of the patients came normal. Moreover, the function of the liver, heart, and kidney continued to be stable.

**Conclusion:**

This report provides an effective regimen of using UCB and UC-MSCs combined with low-dose CsA (1-3 mg/kg/d) to treat PRCA, especially for elderly patients with multiple complications who cannot use the conventional dosage.

## 1 INTRODUCTION

Pure Red Cell Aplasia (PRCA) is a rare disorder that presents with anemia secondary to the failure of erythropoiesis [1]. It is characterized by normocytic, normochromic anemia, associated with reticulocytopenia in the peripheral blood and absent or infrequent erythroblasts in the bone marrow. The pathogenesis is mainly related to the immune mechanism involved in the destruction of red blood cells. The treatment of PRCA mainly includes immunosuppressive therapy (such as cyclosporine A, corticosteroid, and cyclophosphamide), supportive treatments (such as red blood cell transfusion), and plasma exchange [2]. However, the immunosuppressants are cytotoxic, and long-term red cell transfusions also have associated toxicity caused by iron overloading [1, 3]. Refractoriness to immunosuppressive therapy and relapse after successful immunosuppressive therapy are associated with decreased overall survival [4]. For elderly patients, the above treatments have a slow response, a high incidence of infection, and damage to the heart, liver, and kidney functions, which makes it urgent to find new therapeutic regimens. Stem cell therapies for PRCA have been increasingly researched in recent years, but most of them use stem cells derived from bone marrow and peripheral blood, which are very rare from cord blood [5-7]. Cord blood and MSCs have been widely used due to the advantages of easy access, no ethical risk, low risk of infectious diseases, high stem cell content, and immunomodulatory effects [8-10].

In this report, we used UCB and UC-MSCs combined with a low dose of CsA (1-3mg/kg/d) to treat 3 elderly patients with PRCA and achieved good results, providing a new idea for the treatment of PRCA. The criteria for cord blood units were as follows: ABO compatibility of donor and the patient and total nucleated cell (TNC) >1.0×10^7^/Kg. The cell number of UC-MSCs should be more than 7.0×10^7^. UCB was infused first, followed by UC-MSCs after 7 days.

## 2 CASE PRESENTATION (CLINICAL CASES)

**Fig. (1):**
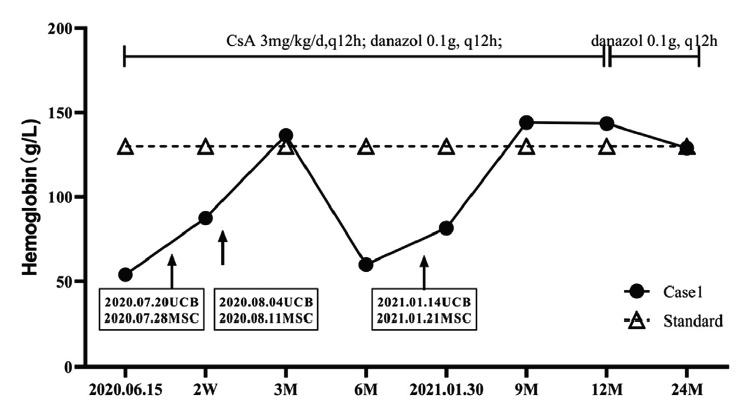
Treatment regimen and changes of HGB in case 1.

**Case 1:** A 97-year-old male was admitted to the hospital on June 15^th^, 2020, due to “fatigue for 4 days”. The patient had a history of hypertension and a space-occupying lesion in the gallbladder. Physical examination showed severe anemia, no jaundice in skin and mucosa, and no petechiae or ecchymosis. Routine blood tests showed that hemoglobin (HGB) was 54g/L and reticulocyte (RET) was 0.4%. Bone marrow biopsy showed active bone marrow hyperplasia, hypoplasia of red blood cells, and dysmorphic changes of late-stage erythroblasts. Chest computed tomography (CT) showed bronchitis with interstitial lesions in the lower lobes of both lungs. Abdominal CT showed gallbladder space-occupying lesion, liver/kidney multiple cysts, left inguinal hernia, prostatic hyperplasia, and calcification. Brain Magnetic Resonance Imaging (MRI) showed multiple ischemic and degenerative infarcts, arteriosclerotic leukoencephalopathy, brain atrophy, and small patches of abnormal signals in the left cerebellar hemisphere, which were consistent with Magnetic Resonance Angiography (MRA) findings of cerebral arteriosclerosis. Electrocardiogram and echocardiography showed frequent premature atrial beats, complete right bundle branch block, aortic valve degeneration with regurgitation (mild), tricuspid regurgitation (mild to moderate), pulmonary hypertension (mild), and reduced left ventricular diastolic function. The patient was diagnosed with PRCA and had multiple complications in the heart, lungs, and renal, without a pathological diagnosis of a space-occupying lesion in the gallbladder due to the patient's age. The treatment of PRCA and changes in HGB are shown in Fig. (**1**). After hospitalization, treatments were given to the patient, including CsA 3mg/kg/d (q12h), danazol 0.1g (q12h), red blood cells 2u (1/qw), and erythropoietin 1.20wu (1/qod), but the curative effect was less apparent after 1 month of treatment. Therefore, on July 20^th^, 2020, one unit of UCB from Shandong Qilu Stem Cell Engineering Co., LTD was infused. The total nucleated cell of the UCB was 1.30×10^9^, of which the number of CD34+ cells was 5.66×10^6^. On July 28^th^, 2020, 7×10^7^ UC-MSCs were infused into the patient. HGB slightly increased to 55g/L, but still less than 60g/L. Given the patient's 97-year-old age and multiple organ complications, 7 days later, a second cell infusion was performed to better promote hematopoietic recovery. The second infusion of UCB was given on August 4^th^, 2020, of which the TNC was 1.509×10^9^, with 4.16×10^6^ CD34+ cells in it. On August 11^th^, 2020, the second infusion of MSC with 8×10^7^ cells was given. About two weeks after the first UCB infusion, HGB and RET recovered to 87.50g/L and 1.8%, respectively, and the patient became independent of blood product transfusion. Three months after the first UCB infusion, HGB returned to 136.5g/L. However, on January 13^th^, 2021, about 6 months after the first UCB infusion, the patient developed anemia again due to an upper respiratory tract infection. Blood routine test showed RBC was 1.75×10^12^/L, HGB was 59.90g/L, and RET was 0.1%. 4U red blood cells were infused into the patient, and the third infusion of UCB was given on January 14^th^, 2021, with 1.063×10^9^ TNC and 1.256×10^7^ CD34+ cells. On January 21^st^, 2021, the third infusion of MSC with 8×10^7^ cells was given. Two weeks after the third UCB infusion, HGB recovered to 81.60g/L, and the patient became independent of blood product transfusion again. When following up at 9 and 12 months after the first UCB infusion, HGB remained normal (Fig. **1**), and at 12 months, CsA was discontinued while danazol 0.1g (q12h) was continued. When following up at 24 months after the first UCB infusion, RBC, HGB, and RET were 3.44×10^12^/L, 129g/L, and 2.3%, respectively, and the patient's liver, heart, and kidney function continued to be stable.

**Case 2:** On March 14^th^, 2022, a 73-year-old male patient was admitted to the hospital due to “fatigue for more than 1 month and anemia for 2 days”, with a history of hypertension and diabetes mellitus. Physical examination showed severe anemia, no jaundiced skin, and no petechiae or ecchymosis. Routine blood tests showed that HGB was 67.4g/L and RET was 0.2%. Chest CT showed chronic inflammation in the left lower lobe and small nodules in both lungs. Bone marrow smear and biopsy showed active proliferation of cells, 89.5% of which were granulocytes, and the percentage of erythroid cells was low, accounting for 1%. There were 67 megakaryocytes, and in some areas, adipocytes and fibrocytes were easily seen. PRCA was diagnosed with hypertension and diabetes mellitus. The treatment of PRCA and changes in HGB are shown in Fig. (**2**). On March 15^th^, 2022, CsA 3mg/kg/d (q12h), Stanozolol 2mg (q12h), and 2u concentrated red blood cells were given to the patient. On March 28^th^, 2022, one unit of UCB was infused, of which the TNC was 1.807×10^9^ and the CD34+ cells were 3.61×10^6^. Then, 7 days later, on April 3^rd^, 2022, one unit of MSC with 7×10^7^ cells was infused. One week after UCB infusion (April 3^rd^, 2023), the patient began to wean off blood products, with RBC 2.32×10^12^/L, HGB 73.9g/L, and RET 0.8%. As shown in Fig. (**2**), the follow-up results of HGB at 3, 6, 9, 12, and 14 months after UCB infusion remained normal. Stanozolol was stopped at 6 months, and the dosage of CsA was reduced to 2mg/kg/d. Then, at 9 months, the dose was reduced to 1.5mg/kg/d, and at 12 months, the dose was reduced to 1mg/kg/d. At the 14-month follow-up, the patient's condition remained stable, and the liver, kidney, and heart function continued to be stable.

**Case 3:** On October 24^th^, 2022, a 66-year-old male patient was admitted to the hospital due to “yellow face, fatigue, and anemia for more than 9 months”. He had type II diabetes mellitus and deep vein valve insufficiency of the lower extremities. Blood routine tests showed HGB and RET were 69g/L and 0.18%, respectively. Bone marrow smear and biopsy showed active proliferation of bone marrow cells, with 89.5% of granulocytes and 1.5% of erythrocytes. The proportion of erythroid cells was low, the mature erythrocytes were normal, and the megakaryocytes were visible. Pure red cell aplastic anemia was diagnosed with type II diabetes mellitus and deep vein valve insufficiency of the lower extremities. The treatment of PRCA and changes in HGB are shown in Fig. (**3**). On October 19^th^, 2021, CsA 1.2mg/kg/d (q12h) and danazol 0.1g (q12h) were given. On October 24^th^, one unit of UCB was infused, of which TNC was 1.915×10^9^, and CD34+ cells were 7.33×10^6^. One unit of MSC was infused on October 31^st^, 2021, with a cell number of 7.5×10^7^. As shown in Fig. (**3**), HGB and RET at 1 week after UCB infusion were 70g/L and 1.17%, and at 3 weeks after UCB infusion, the results came to 87g/L and 1.07%, respectively. HGB at 1, 3, 6, 9, and 12 months after UCB infusion remained normal, and danazol was discontinued at 12 months. The patient's condition continued to be stable, with stable liver, renal, and cardiac function.

## 3 DISCUSSION

PRCA is a highly heterogeneous disease, of which acquired PRCA is mainly caused by direct or indirect attacks of viruses (such as parvovirus B19), drugs (recombinant erythropoietin, rhEPO), antibodies (autoantibodies IgG), myeloid mutations, or immune cells (αβ / γδT, NK/T) on erythroid progenitor cells, inhibiting the proliferation, differentiation and maturation of erythroid cells [11-15]. CsA is the first choice for the treatment of acquired PRCA, with an effective rate of up to 75%. The recommended dosage is 6 mg/kg/d [3, 16]. However, CsA has toxic effects, including nephrotoxic, hepatotoxic, neurotoxic, and cardiotoxic effects [17]. The other immunosuppressants also have disadvantages, of which corticosteroid (CX) can cause side effects, such as infection, hyperglycemia, and osteoporosis [18], while cyclophosphamide (CTX) can cause bone marrow suppression, liver function damage, and other adverse reactions [19]. Long-term use of immunosuppressants is prone to co-infection, especially fungal, opportunistic pathogens, and viruses, while long-term red cell transfusions also have iron overloading [1, 3]. In our report, the 3 elderly patients were treated with a low dose of CsA (1-3mg/kg/d), and the effect was as good as the recommended dosage, with no liver, renal, or cardiac function injury occurring during the follow-up, which verified the effectiveness of low-dose CsA combined with UCB and MSC treatment.

UCB is not only widely used for Hematopoietic Cell Transplantation (HCT) but can also be used as a substitute for transfusion and novel non-HCT cell therapy materials [20-22]. Allogeneic cord blood transfusion has been successfully used in anemia [23, 24], refractory immune cytopenia [25], ischemic stroke [26], autism spectrum disorder [27], *etc*. Cases of PRCA treatment with UCB have also been reported [5, 25, 28, 29]. Hu, Q.L., *et al*. verified the safety and efficacy of using two units of allogeneic unrelated non-HLA matched umbilical cord blood transfusion in 4 cases of PRCA patients [25]. Additionally, UCB is less prone to PRCA than other stem cells [6], which may be due to the fact that UCB is rich in various stem cells, less mature cells, lower immunogenicity, and fewer viruses/antibodies. Moreover, previous results showed that cord blood infusion in severe aplastic anemia patients led to more rapid recovery, and no donor stem cell engraftment occurred, indicating that UCB plays a role in hematopoietic support [30-32].

MSCs and the derived exosomes have been widely used in hematological diseases, acute respiratory distress syndrome, kidney diseases, graft-*versus*-host disease, osteoarthritis, stroke, Alzheimer’s disease, and type 1 diabetes [33-40]. MSCs play roles mainly due to their functions of immune regulation through paracrine. MSCs could regulate the immune function of innate and adaptive immune cells by modulating the activation, expansion, and differentiation of natural killer cells, dendritic cells, B lymphocytes, macrophages, and T lymphocytes [41-49]. As for the direct therapeutic effect of MSC on PRCA, Vera *et al*. reported a case of chronic PRCA caused by ABO-incompatible allogeneic transplantation, which was rapidly recovered by MSC infusion [50]. Based on studies on the pathogenesis of PRCA and the role of MSCs in hematological and immune system diseases, we speculated that MSCs may affect the patients through their immunomodulatory effect.

In our report, the 3 patients were all elderly men, and after infusion of UCB and MSC, they were all independent of blood product transfusion within 1-2 weeks, with hemoglobin and other indicators remaining within the normal range during the follow-up from 3 months to 1 year. Our results supported the previous reports, which implied that the effect of stem cells to cure PRCA may be related to the hematopoietic support function of UCB and the immunomodulatory role of MSC. However, the mechanism needs to be further studied. Moreover, patients with PRCA who receive HCT at a younger age have a better prognosis than those who receive HCT at an older age or after chronic blood transfusion [51]. In our report, the time from CBU infusion to free from blood product transfusion of case 2 (aged 73 years) was 1 week, which was significantly shorter than the 2 weeks of case 1 (aged 97 years), which is consistent with the above reports. PRCA cannot be evaluated in large controlled clinical trials due to the rarity of the disease. Therefore, most recommendations regarding this disease are based on retrospective trials or case reports.

The innovation of our case report mainly includes the following two points: Firstly, we used a low dose of CsA (1-3mg/kg/d) for elderly patients, which was much lower than the recommended dose of 6 mg/kg/d, effectively reducing the toxicity of CsA. Secondly, the regimen of UCB combined with UC-MSCs infusion and CsA could fully play the hematopoietic recovery function of UCB and the immune regulation function of UC-MSCs. The treatment effect in all three patients was very obvious, demonstrating the safety and efficacy of this regimen. All the patients in this study were satisfied with the treatments they received.

## CONCLUSION

This report provides an effective regimen of using UCB and UC-MSCs combined with a low dose of CsA (1-3 mg/kg/d) to treat PRCA, especially for elderly patients with multiple complications who cannot use the conventional dosage.

## Figures and Tables

**Fig. (2):**
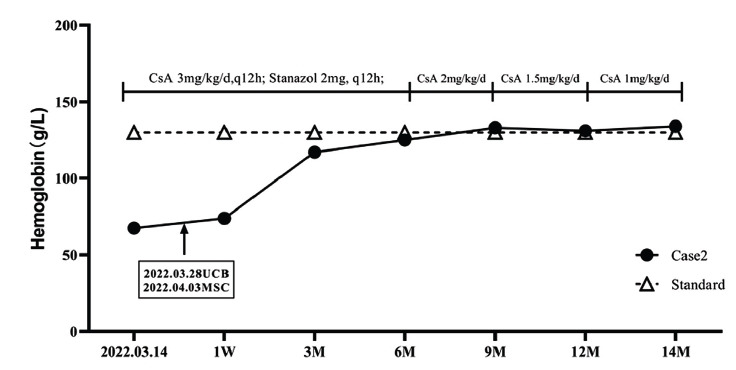
Treatment regimen and changes of HGB in case 2.

**Fig. (3):**
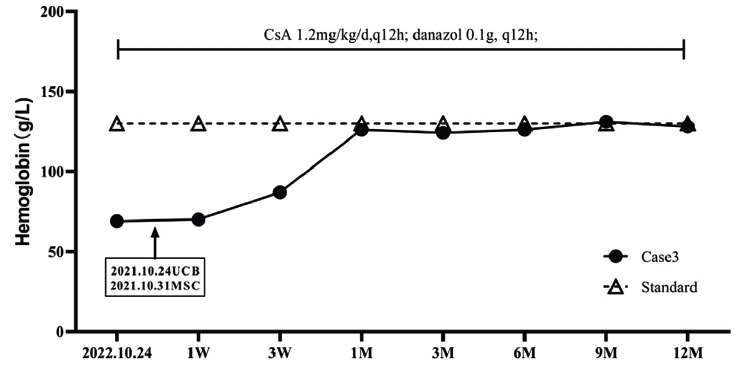
Treatment regimen and changes of HGB in case 3.

## Data Availability

The data and supportive information are available within the article.

## LIST OF ABBREVIATIONS

CT: Computed Tomography
HCT: Hematopoietic Cell Transplantation
MRA: Magnetic Resonance Angiography
MRI: Magnetic Resonance Imaging
PRCA: Pure Red Cell Aplasia
TNC: Total Nucleated Cell
UC-MSCs: Umbilical Cord Mesenchymal Stem Cells

## STANDARDS OF REPORTING

CARE guidelines were followed.
